# Percutaneous transhepatic and endoscopic biliary drainage for malignant biliary tract obstruction: a meta-analysis

**DOI:** 10.1186/1477-7819-12-272

**Published:** 2014-08-23

**Authors:** Jian-jun Leng, Ning Zhang, Jia-hong Dong

**Affiliations:** 1 Institute of Hepatobiliary Surgery, Chinese PLA General Hospital, 28 Fuxing Road, Beijing, 100853 China; 2 Department of Hepatobiliary Surgery, Xijing Hospital, The Fourth Military Medical University, 15 Changle West Road, Xi’an, Shannxi 710032 China

**Keywords:** Biliary tract neoplasms, Biliary tract surgical procedures, Cholangiocarcinoma, Endoscopic biliary drainage, Extrahepatic biliary ducts, Malignancy-induced biliary obstruction, Obstructive jaundice, Percutaneous transhepatic biliary drainage

## Abstract

**Background:**

Various malignant tumors can obstruct the extrahepatic biliary tract. Two major techniques for restoring bile flow in this circumstance are endoscopic biliary drainage (EBD) and percutaneous transhepatic biliary drainage (PTBD).We conducted a meta-analysis to compare the effectiveness and safety of the two techniques.

**Methods:**

Medline, EMBASE and the Cochrane Library database were searched for articles published between January 1980 and December 2013. The outcome measures were therapeutic success rate (primary), 30-day mortality rate and overall complications.

**Results:**

Of 264 screened articles, 3 randomized controlled trials comprising an aggregate total of 183 cancer patients were included in the meta-analysis. Our analysis showed no significant difference in restoration of bile flow between patients treated with EBD and those treated with PTBD (odds ratio (OR) = 2.34, 95% confidence interval (CI) = 0.32 to 17.16, *P* = 0.401). However, the result of sensitivity analysis indicated that the study conducted by Speer *et al*. influenced the pooled estimates. After the Speer *et al*. study was excluded, the therapeutic success rate of patients treated with PTBD was significantly greater than that of those who underwent EBD (OR = 5.48, 95% CI: 2.26 to 13.28, *P* < 0.001). The 30-day mortality and complication rates were similar in the EBD and PTBD groups.

**Conclusions:**

The results of our meta-analysis indicate that PTBD had a higher therapeutic success rate than EBD in the treatment of malignancy-induced biliary obstruction. The mortality and complication rates of the two techniques were similar.

**Electronic supplementary material:**

The online version of this article (doi:10.1186/1477-7819-12-272) contains supplementary material, which is available to authorized users.

## Background

Malignant tumors that obstruct bile flow usually carry a very poor prognosis [1]. Tumor-induced obstructive jaundice can be caused by Klatskin tumors (hilar cholangiocarcinoma), pancreatic adenocarcinoma, gallbladder carcinoma, metastases in theporta hepatis lymph nodes, distal cholangiocarcinoma orhepatocellular carcinoma (HCC) [2–5]. Jaundice occurs in 5% to 44% of patients withHCC [3]. Symptoms include jaundice and pruritus, which can significantly impair patients’ quality of life. The major goal of palliative treatment for obstructing biliary tumors is the restoration of bile flow to the intestine [1]. Median survival has been found to be significantly longer in patients with restored bile drainage (4.8 to 11.8 months), regardless of technique, than in patients with failed attempts at biliary drainage (1.3 to 1.8 months) [3, 6, 7]. Restoration of bile drainage also improves the patient’s quality of life.

Treatment modalities that can restore adequate bile duct drainage in malignant biliary obstruction cases include surgical biliary bypass, endoscopic biliary drainage (EBD) and percutaneous transhepatic biliary drainage (PTBD) [1–15]. None of these procedures has been proven superior to the others, and the most effective procedure for decompression of bile obstruction remains controversial. Cholangitis and pancreatitis are common complications of all the biliary decompression procedures [8].

It has been difficult to compare the effectiveness and safety of surgical bypass to endoscopic stenting in the treatment of malignant biliary duct obstruction, because many variables can influence the results: heterogeneity of patient populations studied, variations in tumor type, improvement in endoscopic and surgical techniques and differences in study design [2–4, 6–8, 12–15]. Nonetheless, the results of some comparative studies are available.Randomized clinical trials (RCTs) have shown no significant difference in overall survival rates between patients who were treated with endoscopic stenting and those who underwent surgical bypass [9–11]. However, stenting was associated with a significantly lower early complication rate [10] and shorter initial hospital stay [11], whereas the surgical bypass group had a significantly lower late complication rate [10]. The comparative results of the EBD and PTBD procedures in RCTs have varied from a significant benefit for the EBD procedure [12] to a significant benefit for the PTBD technique [4, 13].

The comparative effectiveness of EBD and PTBD in the treatment of the various types of malignancy that can obstruct the bile duct remains unclear. Therefore, we conducted a meta-analysis of studies published through January 1980 and December 2013 to try to clarify this issue.

## Methods

This article is in accordance with the PRISMA (Preferred Reporting Items for Systematic Reviews and Meta-Analysis) guidelines [16]. This study doesn’t involve human subjects and does not require IRB review or consent. The population, intervention, comparator, outcomesand study design (PICOS) components are as follows: P: patients with malignant biliary tract obstruction; I: percutaneous transhepatic biliary drainage; C: endoscopic biliary drainage; OS: therapeutic success rate, 30-day mortality rate and complications.

### Search strategy and data sources

Two reviewers independently searched Medline, EMBASE, Cochrane Library (CENTRAL), from January 1980 to December 2013, for articles with these keywords: malignant biliary obstruction, percutaneous endoscopic biliary drainage, cholangiocarcinoma, Klatskin tumor, duodenal cancer, ampullary cancer, pancreatic cancer and hepatocellular carcinoma. Searches were conducted using these specific keyword combinations: percutaneous AND malignant biliary obstruction; endoscopic AND malignant biliary obstruction; and percutaneous AND endoscopic AND biliary drainage.

### Study selection

Inclusion criteria in the meta-analysis were original RCTs that compared the efficacy and safety of PTBD and EBD used for treatment of malignant biliary obstruction. The meta-analysis excluded review articles, case reports, retrospective studies, cohort studies, single-arm prospective studies, letters, comments, editorials, non-English-language publications and trials of patients with biliary obstruction that was not caused by malignancy. Two reviewers (LJJ and ZN) screened and selected the articles independently. In case of disagreement on inclusion of an article, a third reviewer (DJH) was consulted.

### Data extraction and quality assessment

The reviewers extracted the following information in a blinded manner from the included articles: the number of cases, patients’ ages (in years as a continuous variable), sex (male or female), type of stent placed (plastic or metal stent), follow-up period (in months as a continuous variable), type of malignancy (carcinoma of the gallbladder, primary carcinoma of the pancreas or bile ducts), use of prophylactic antibiotics (types of antibiotics), therapeutic success rate (successful drainage in the short term), 30-day mortality rate (in percentage), complications (overall, cholangitis and pancreatitis) and reference citation. All extracted data were checked by a third reviewer (DJH). The primary outcome for this study was the therapeutic success rate. Secondary outcomes were 30-day mortality rates and overall complication rates.

Two reviewers (LJJ and ZN) assessed the risk of bias in the studies, as described in the Cochrane Handbook for Systematic Reviews of Interventions (version 5.1.0) [17]. They assigned a low risk, high risk or unclear risk to the attributes of each included article: (1) random sequence generation, (2) allocation concealment, (3) blinding (patients, personnel and assessor), (4) adequate assessment of each outcome, (5) avoidance of selective outcome reporting and (6) intention-to-treat analysis.

### Statistical analysis

Odds ratios (ORs) with 95% confidence intervals (CIs) were calculated for binary outcome data and were compared between PTBD and EBD groups. A χ^2^test was used to assess the presence of heterogeneity across studies, and *I*^2^ was used to assess the degree of heterogeneity. An OR >1 for the therapeutic success rate indicated that PTBD was favored. In contrast, an OR <1 for the 30-day mortality rate and overall complication rate indicated that PTBD was favored. If heterogeneity existed between studies (Q-statistic with *P* < 0.1 [18] or *I*^2^ > 50% [19]), we used the random-effects model (DerSimonian-Laird method) [20]. Otherwise, the fixed-effects model was recommended (Mantel-Haenszel method). Statistical significance was set at a two-sided *P*-value <0.05. The small number of the selected studies negated assessment of publication bias by the funnel plot method [21]. Sensitivity analysis was performed for primary outcomes based on the leave-one-out approach. All analyses were performed with Comprehensive Meta-Analysis statistical software, version 2.0 (Biostat, Englewood, NJ, USA).

## Results

### Identification of relevant studies and risk of bias

**Figure 1:**
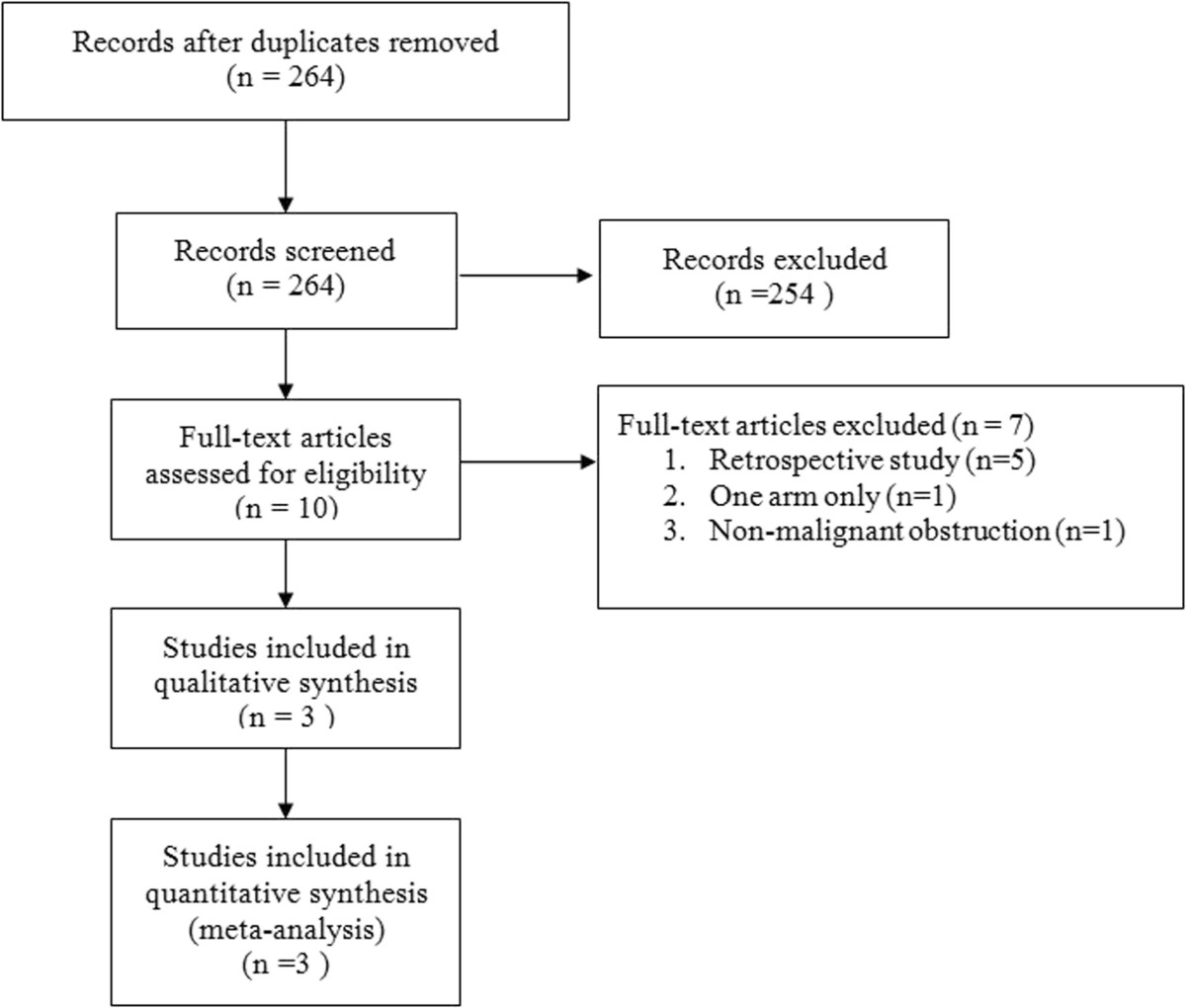
**Flowchart for selection of articles for the meta-analysis.**

**Figure 2:**
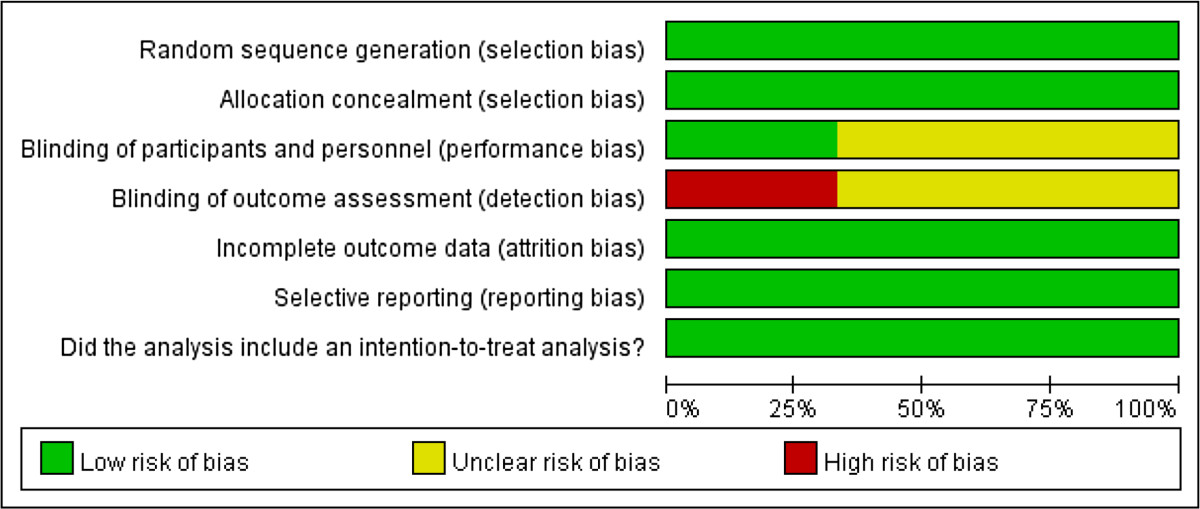
**The risk of bias assessments.**

Initially, 482 articles were identified (Medline, 252; EMBASE, 200; Cochrane Library, 30). The flowchart for the selection of studies is shown in Figure 1. The reviewers excluded 264 articles describing 254 studies identified in the literature search. Three of the ten full-text articles described RCTs and met all the inclusion criteria [4, 12, 13]. The reasons for exclusion of other full-text articles are given in the flowchart. In addition, the risk of reporting bias is illustrated in Figure 2.

### Description of studies

The three RCTs included anaggregate total of 183 participants (range, 54 to 75 participants), with 92 patients treated with EBD and 91 patients treated with PTBD (Table 1). Saluja *et al*. [13] compared the two modalities in patients with carcinoma of the gallbladder, whereas Speer *et al*. [12] and Pinot *et al*. [4] compared them in patients with carcinoma of the pancreas, bile ducts or gallbladder [4, 12]. Plastic stents were tested in 129 patients [12, 13] and self-expanding metal stents in 54 patients [4]. The definition of therapeutic success ranged from a 20% decline [4, 12] to a 50% decline [13] of serum bilirubin values from baseline (Table 2).

**Table 1:** **Summary of studies included in the meta-analysis** ^**a**^

First author	Year	Study type	Comparison	Number of cases	Malignancy causing biliary obstruction	Age, yr	Males,%	Type of stent placed	Prophylactic antibiotics	Follow-up period
Saluja	2008	RCT	PTBD vs. EBD	27 vs. 27	Carcinoma of the gallbladder	51 vs. 50	37% vs. 30%	Plastic stent	Cefoperazone + sulbactam	3 months
Piñol	2002	RCT	PTBD vs. EBD	28 vs. 26	Primary carcinoma of the pancreas, gallbladder, or bile ducts, or to regional lymph node metastases	75 vs. 70	43% vs. 42%	Metal stent	Ciprofloxacin	Median: 2.5 months
Speer	1987	RCT	PTBD vs. EBD	36 vs. 39	Primary carcinoma of the pancreas, gallbladder, or bile ducts	73 vs. 72.5	NA	Plastic stent	NA	NA

^a^EBD, Endoscopic biliary drainage; NA, not available; PTBD, Percutaneous transhepatic biliary drainage; RCT, Randomized controlled trial.

**Table 2:** **Summary of primary and secondary outcomes and complications**

First author	Year	Definition of therapeutic success	Therapeutic success^a^,%	30-day mortality^a^,%	Overall complications^a^,%	Incidence of cholangitis^a^,%	Incidence of pancreatitis^a^,%
Saluja	2008	Bilirubin declined to <50% of the pretreatment value within 7 days after drainage	89 vs. 41^b^	4 vs. 8	18 vs. 52^b^	11 vs. 48^b^	0 vs. 3.7
Piñol	2002	Bilirubin declined by ≥ 20% of the pretreatment value	71 vs. 42^b^	36 vs. 42	61 vs. 35	NA	0 vs. 3.8
Speer	1987	Bilirubin declined by ≥20% of the pretreatment value during the initial admission	61 vs. 81	33 vs.15	67 vs. 19^b^	13.9 vs. 17.9	NA

^a^Percutaneous transhepatic biliary drainage vs. endoscopic biliary drainage. ^b^Significant difference between PTBD and EBD. NA, not available.

### Primary outcome: therapeutic success rate

**Figure 3:**
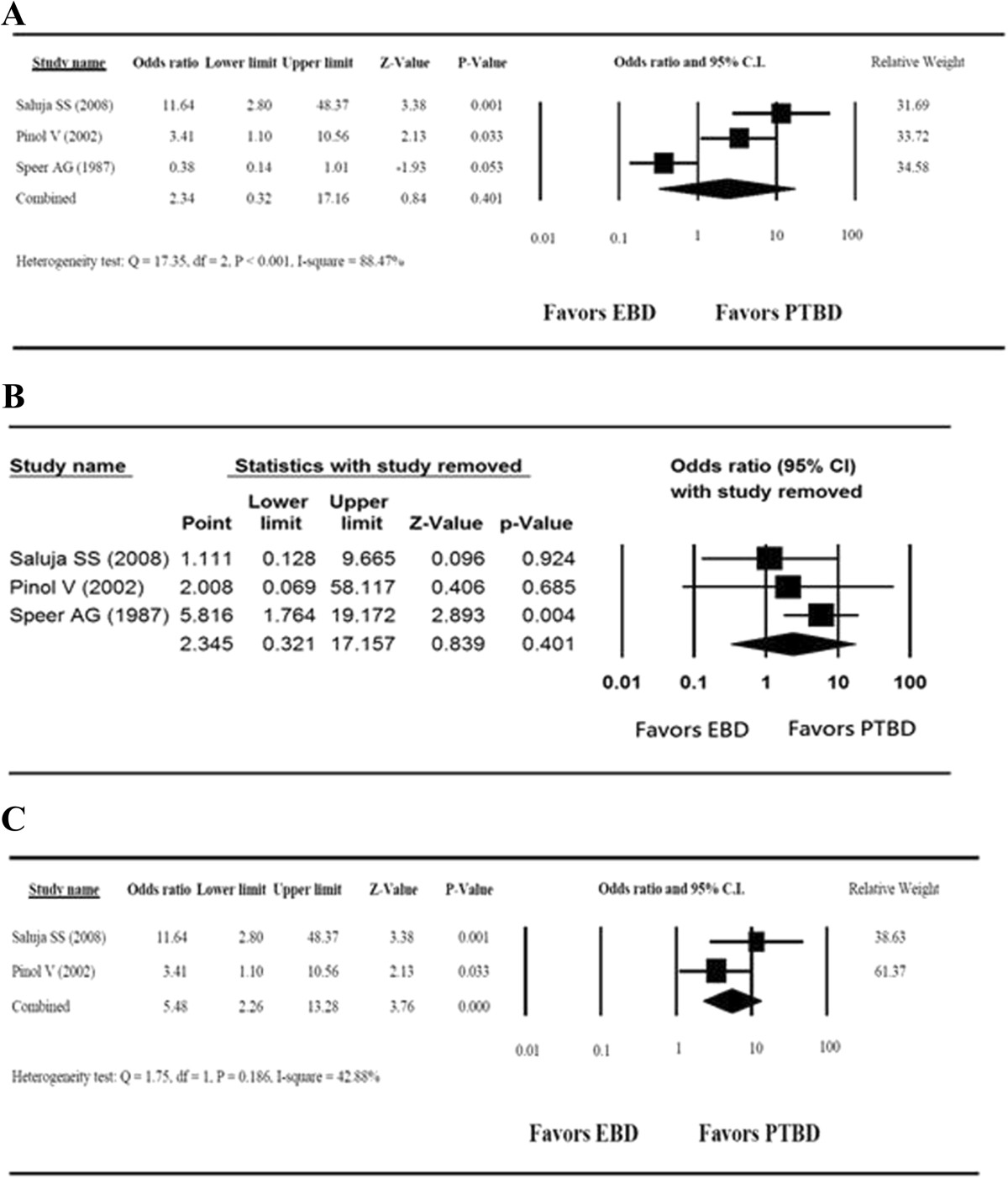
**Forest plots for therapeutic success rates of the three randomized controlled trials. (A)** Random-effects analysis of overall success rates. **(B)** Sensitivity analysisof primary outcomes based on the leave-one-out approach. **(C)** Success rates for the two randomized controlled trials after excluding the Speer *et al*. trial. CI, Confidence interval; EBD, Endoscopic biliary drainage; PTBD, Percutaneous transhepatic biliary drainage.

Analysis of the pooled data from the three studies indicated a high heterogeneity in the therapeutic success rates (Q = 17.35, *I*^2^ = 88.47%, *P* < 0.001); therefore, we used a random-effects model of analysis. The OR revealed no significant difference in therapeutic success rates between the PTBD and EBD groups (overall OR = 2.34, 95% CI = 0.32 to 17.16, *P* = 0.401) (Figure 3A). Sensitivity analysis was performed for primary outcomes based on the leave-one-out approach (Figure 3B). The results indicated that the study conducted by Speer *et al*. [12] influenced the pooled estimates. After those results were excluded, the heterogeneity was substantially decreased (Q = 1.75, *I*^2^ = 42.88%, *P* = 0.186), and the results then indicated that the PTBD group had a significantly higher therapeutic success rate than did the EBD group (OR = 5.48, 95% CI = 2.26 to 13.28, *P* < 0.001) (Figure 3C).

### Secondary outcome: 30-day mortality

**Figure 4:**
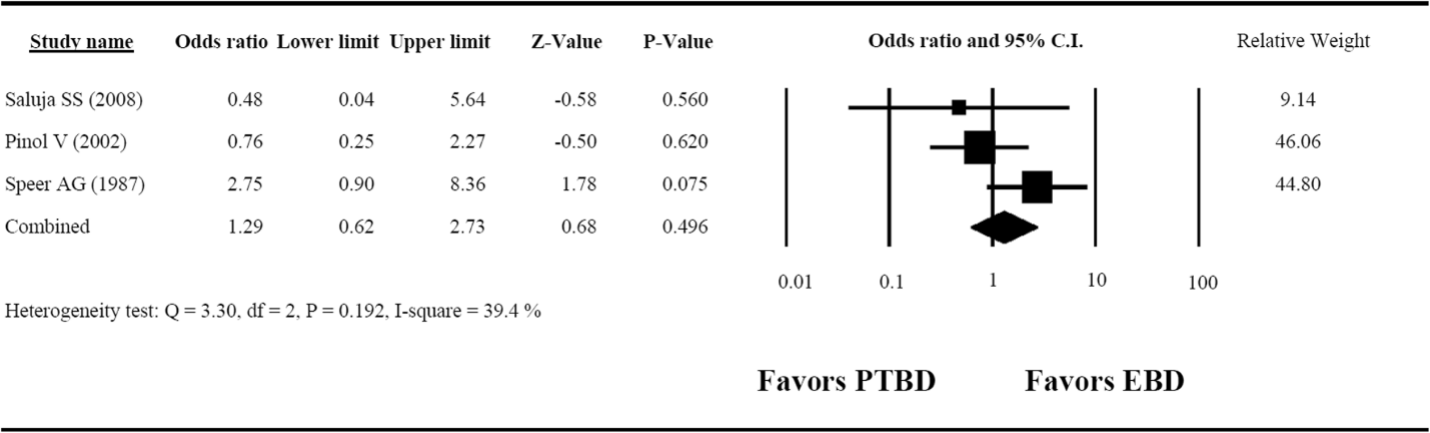
**Forest plot for 30-day mortality.** CI, Confidence interval; EBD, Endoscopic biliary drainage; PTBD, Percutaneous transhepatic biliary drainage.

The heterogeneity of the 30-day mortality data was not significant (Q = 3.30, *I*^2^ = 39.4%, *P* = 0.192) according to the fixed-effects model of analysis. The overall OR was 1.29 (95% CI = 0.62 to 2.73, *P* = 0.496) (Figure 4) and did not reveal a significant difference in 30-day mortality between the PTBD and EBD groups.

### Incidence of overall complications

**Figure 5:**
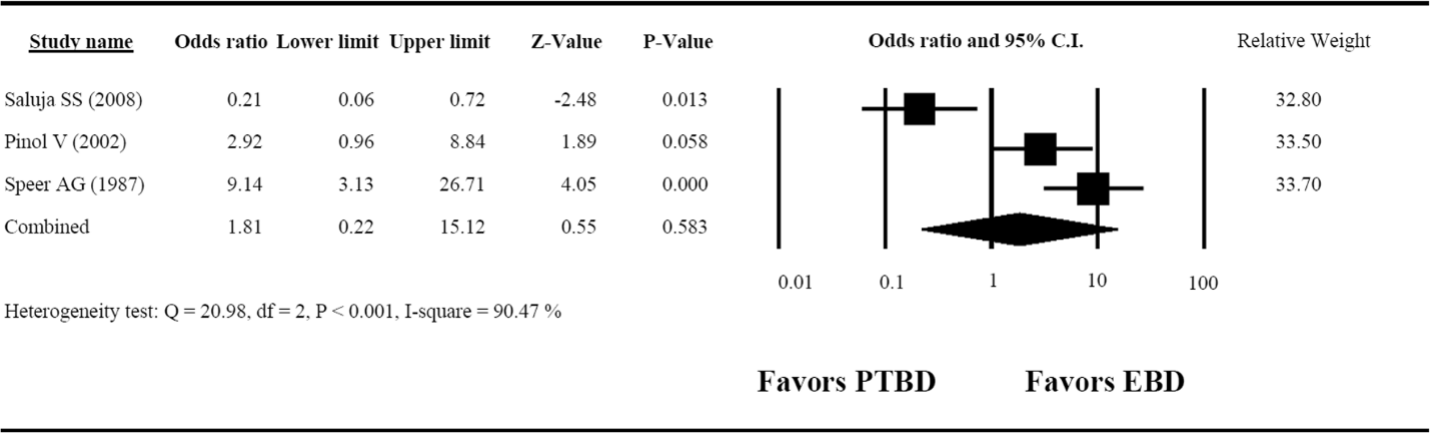
**Forest plot for the overall rate of complications.** CI, Confidence interval; EBD, Endoscopic biliary drainage; PTBD, Percutaneous transhepatic biliary drainage.

The incidence of overall complications in the studies had high heterogeneity (Q = 20.98, *I*^2^ = 90.47%, *P* < 0.001). The overall OR = 1.81 (95% CI = 0.22 to 15.12, *P* = 0.583) (Figure 5), as calculated using a random-effects model of analysis. The OR revealed no significant difference in the incidence of overall complications between the PTBD and EBD groups.

## Discussion

In this meta-analysis, we found no significant difference in the therapeutic success rate, 30-day survival rate or rate of complications between patients treated with EBD and those treated with PTBD for malignant biliary obstruction in three RCTs reviewed [4, 12, 13]. However, sensitivity analysis was performed for primary outcomes based on the leave-one-out approach, with the therapeutic response in two of the studies was separately calculated. That revision revealed a significantly higher therapeutic response for PTBD than for EBD, although there was much heterogeneity in the reported success rates.

The heterogeneity could have resulted from various factors, such as variability in the types of stents (self-expanding metal [4] vs. plastic [12, 13]) and instrumentation used, causes of the biliary obstructionand location and extent of the tumors. The lower success rate of PTBD in the study of Speer *et al*. [12] may have been related to the use of less-advanced instrumentation. In the study of Garcarek *et al*. [20], a significantly lower rate of pericapsular bile leak in procedures performed from 2000 to 2006 compared to 2007 and 2011 may have been due to the use of novel instrumentation in the latter period [22]. Self-expanding metal stents have several advantages over plastic stents, including larger internal diameter, lower risk for stent occlusion, need for additional surgical interventions, and relatively long patency (about 10 months) [23]. The various causes of biliary obstruction described in the studies were cancers of the gallbladder [4, 12, 13], pancreas [4, 12] and bile ducts [4, 12], as well as metastases [4]. Another technical consideration is that one or the other of the two techniques may be more efficacious for treatment of a specific type of tumor, such as hilar cholangiocarcinoma. In this regard, hilar cholangiocarcinoma (Klatskin tumor) was the cause of biliary obstruction in four of the five trials [2, 6, 8, 15, 24], and PTBD had a higher therapeutic success rate than EBD in those. The rates of complication with the two treatments were either similar [2, 6] or lower [8, 15] with PTBD.

With respect to the possible influence of location of the tumor and its extent on the therapeutic success of the drainage technique, tumor infiltration in the second portion of the duodenum and major papilla may prevent cannulation of the bile duct during EBD. In that case, an alternative strategy, such as surgical bypass or PTBD [25], may be needed. Also, patients with proximal biliary obstruction may have a higher incidence of cholangitis and bacteribilia than do those with distal biliary obstruction [26].

The reported incidence of early cholangitis associated with PTBD and EBD ranges from 11% to 48% [12, 13]. Multivariate logistic regression analysis identified the following statistically significant predictive factors for the immediate development of cholangitis after PTBD: history of cholangitis, recent biliary drainage (≤6 months), elevated C-reactive protein and low serum albumin concentration [5]. In one study of PTBD-treated patients [5], the presence of fever was significantly associated with increased the risk for bacteremia and shock, leading the authors to suggest that prophylactic antibiotics should be administered to patients undergoing PTBD.

Our meta-analysis has some limitations. First, relatively few RCTs have been conductedin which investigators compared the outcomes and complication rates of cancer patients treated with PTBD or EBD. Second, both Speer *et al*. [12] and Piñol*et al*. [4] used relatively less stringent criteria for therapeutic success (20% decline in serum biliary concentration), in contrast to the 50% decline in the study of Saluja *et al*. [13]. As mentioned above, the diverse array of tumors and their locations may also have affected the heterogeneity of our meta-analyses. Third, language bias is present because the studies published in languages other than English were excluded. Fourth, technical advances in intensive care made during the period of the studies could have affected the results.

## Conclusions

The results of our meta-analysis indicate that PTBD had a higher therapeutic success rate than EBD in the treatment of malignancy-induced biliary obstruction. We found that the mortality and complication rates of the two techniques were similar.

## Authors’ original submitted files for images

Below are the links to the authors’ original submitted files for images. Authors’ original file for figure 1 Authors’ original file for figure 2 Authors’ original file for figure 3 Authors’ original file for figure 4 Authors’ original file for figure 5
