# Supplementary figures and images for: Percutaneous transhepatic and endoscopic biliary drainage for malignant biliary tract obstruction: a meta-analysis
Source: World J Surg Oncol. 2014 Aug 23;12:272. doi: 10.1186/1477-7819-12-272 (PMC6389255; doi:10.1186/1477-7819-12-272)

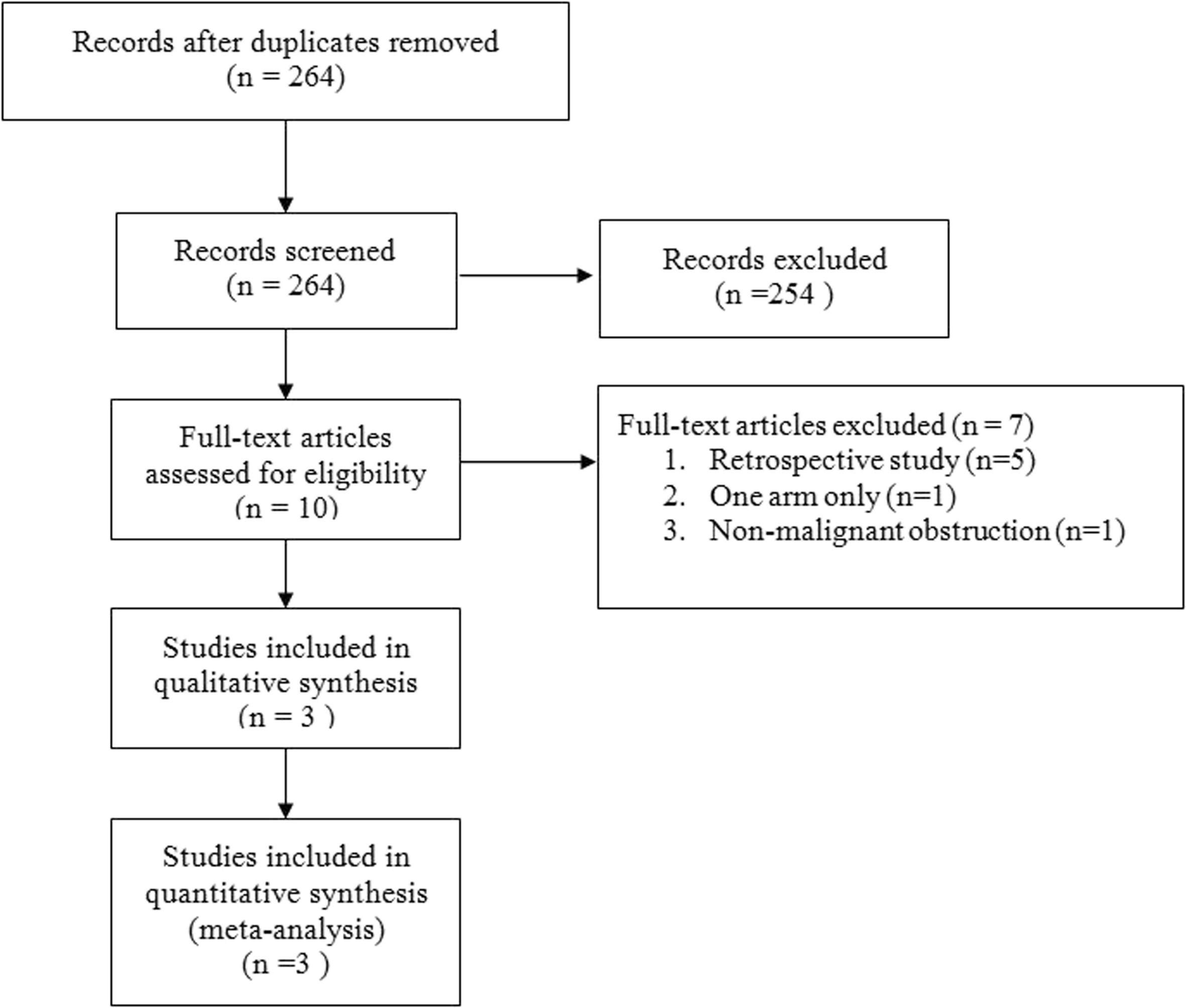

Supplement: Supplementary file 1 — Authors’ original file for figure 1 [file 12957_2014_1936_MOESM1_ESM.tif]

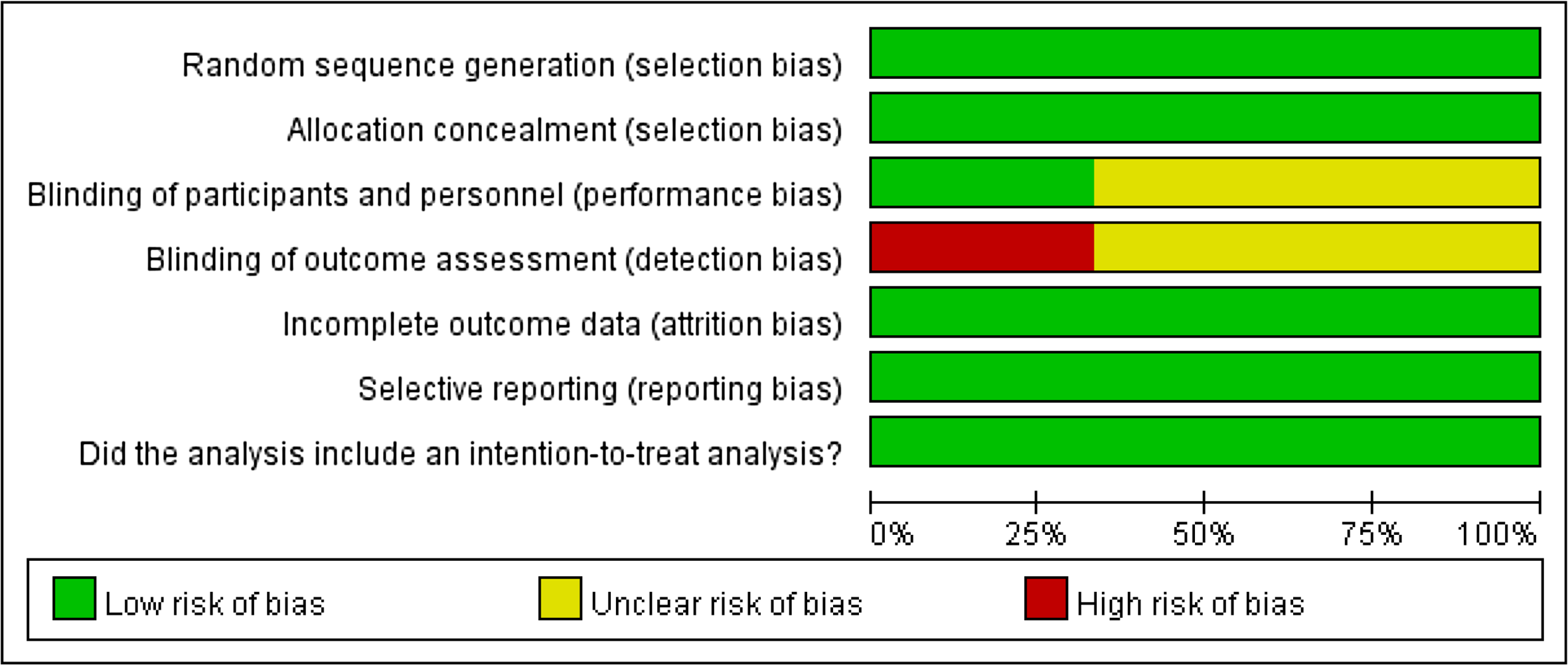

Supplement: Supplementary file 2 — Authors’ original file for figure 2 [file 12957_2014_1936_MOESM2_ESM.tif]

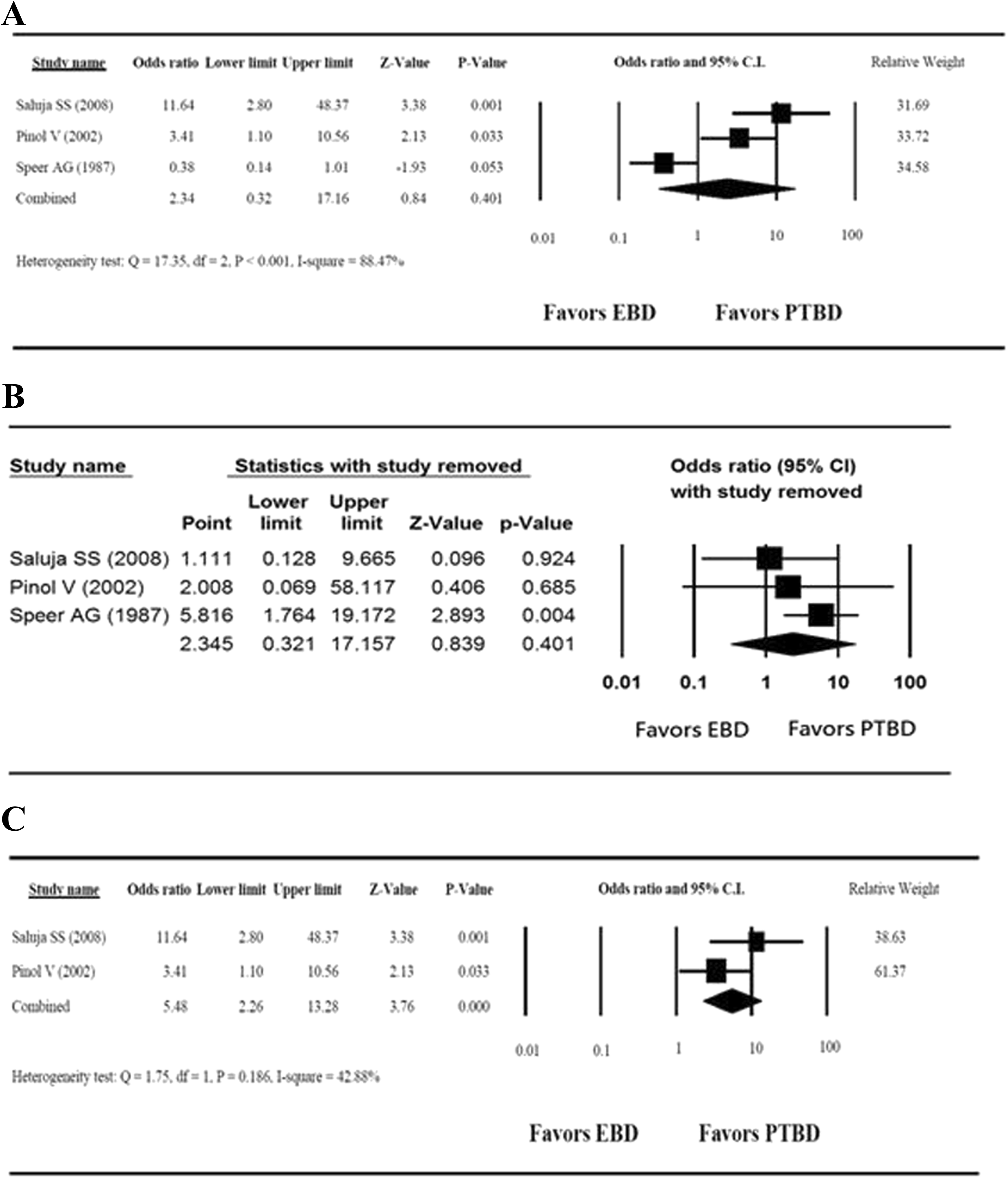

Supplement: Supplementary file 3 — Authors’ original file for figure 3 [file 12957_2014_1936_MOESM3_ESM.tif]

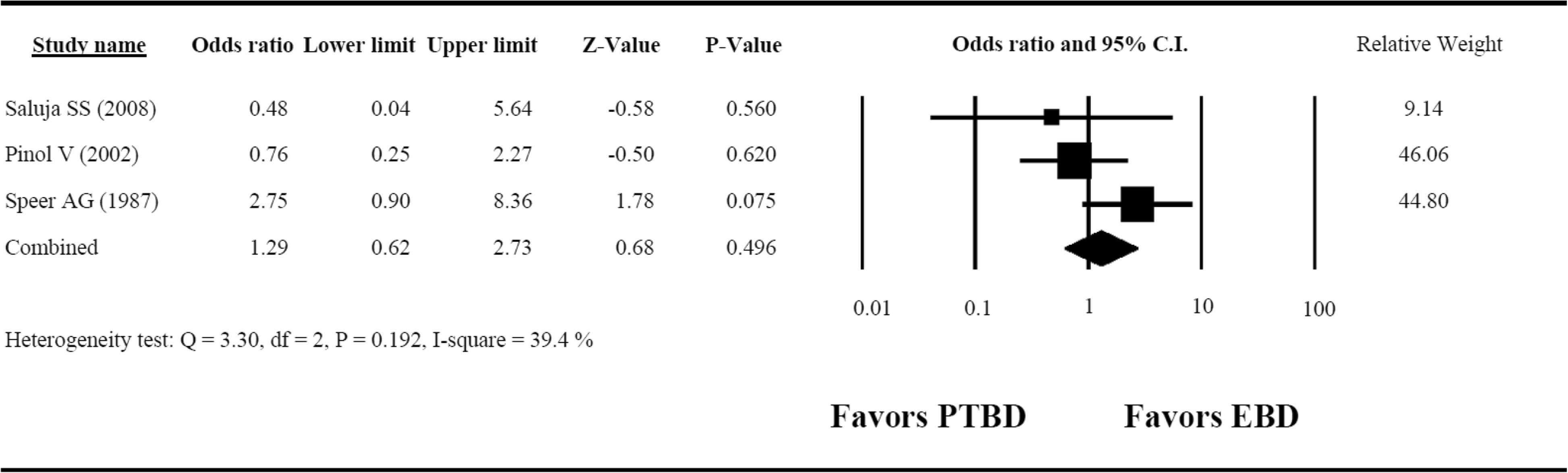

Supplement: Supplementary file 4 — Authors’ original file for figure 4 [file 12957_2014_1936_MOESM4_ESM.tif]

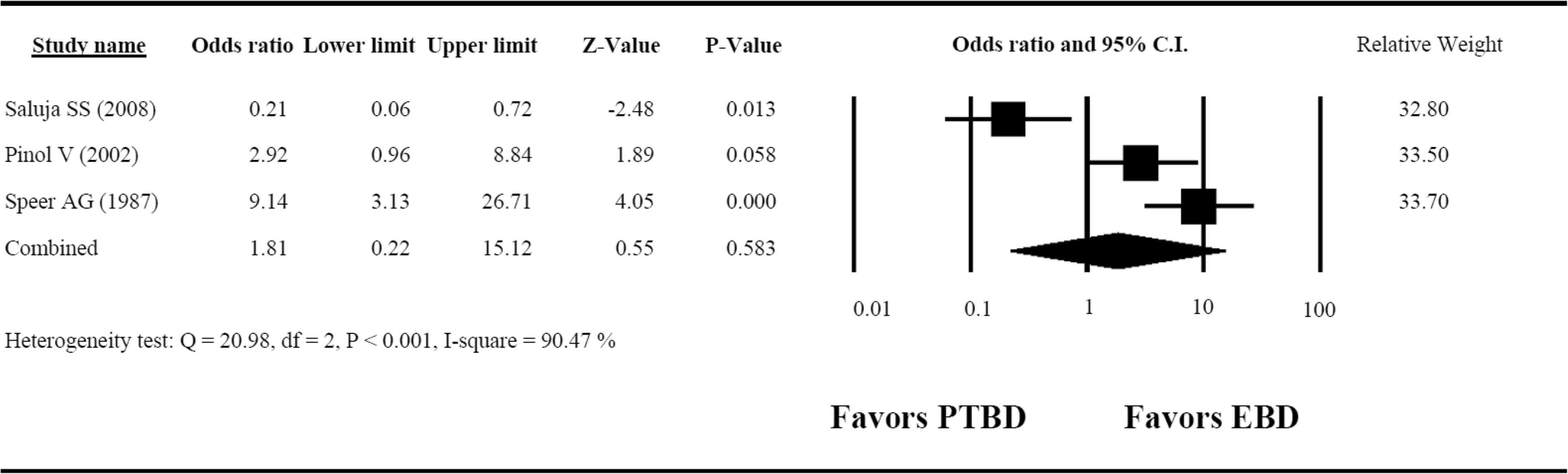

Supplement: Supplementary file 5 — Authors’ original file for figure 5 [file 12957_2014_1936_MOESM5_ESM.tif]
